# *Brevibacterium* from Austrian hard cheese harbor a putative histamine catabolism pathway and a plasmid for adaptation to the cheese environment

**DOI:** 10.1038/s41598-019-42525-y

**Published:** 2019-04-16

**Authors:** Justin M. Anast, Monika Dzieciol, Dylan L. Schultz, Martin Wagner, Evelyne Mann, Stephan Schmitz-Esser

**Affiliations:** 1 Interdepartmental Microbiology Graduate Program Iowa State University, Ames, IA USA; 2 Department of Animal Science, Iowa State University, Ames, IA USA; 3 Institute for Milk Hygiene, University of Veterinary Medicine Vienna, Vienna, Austria; 4 Interdepartmetal Microbiology Undergraduate Program, Iowa State University, Ames, IA USA; 5 Austrian Competence Center for Feed and Food Quality, Safety and Innovation (FFoQSI), Technopark C, 3430 Tulln, Austria

## Abstract

The genus *Brevibacterium* harbors many members important for cheese ripening. We performed real-time quantitative PCR (qPCR) to determine the abundance of *Brevibacterium* on rinds of Vorarlberger Bergkäse, an Austrian artisanal washed-rind hard cheese, over 160 days of ripening. Our results show that *Brevibacterium* are abundant on Vorarlberger Bergkäse rinds throughout the ripening time. To elucidate the impact of *Brevibacterium* on cheese production, we analysed the genomes of three cheese rind isolates, L261, S111, and S22. L261 belongs to *Brevibacterium aurantiacum*, whereas S111 and S22 represent novel species within the genus *Brevibacterium* based on 16S rRNA gene similarity and average nucleotide identity. Our comparative genomic analysis showed that important cheese ripening enzymes are conserved among the genus *Brevibacterium*. Strain S22 harbors a 22 kb circular plasmid which encodes putative iron and hydroxymethylpyrimidine/thiamine transporters. Histamine formation in fermented foods can cause histamine intoxication. We revealed the presence of a putative metabolic pathway for histamine degradation. Growth experiments showed that the three *Brevibacterium* strains can utilize histamine as the sole carbon source. The capability to utilize histamine, possibly encoded by the putative histamine degradation pathway, highlights the importance of *Brevibacterium* as key cheese ripening cultures beyond their contribution to cheese flavor production.

## Introduction

Production of cheese has been documented in many different world cultures dating back more than 7500 years^1,2^. The production of cheese is dependent on complex interactions of diverse microorganisms dispersed throughout the cheese ingredients and the production facility. Particularly in long-ripened cheeses, the microorganisms on the cheese surface contribute significantly to flavor production. These cheese rind microbial communities can either be inoculated artificially with surface-ripening cultures during the manufacturing process, be present in starting ingredients, or establish themselves through inoculation from the microbial communities of the ripening cellar environment during the ripening process^3–6^. Many genera of the bacterial phylum *Actinobacteria,* including – among others - the genus *Brevibacterium,* are important for flavor production during cheese ripening^5,7–9^. The contribution of *Brevibacterium* towards cheese production has been under investigation for some time, showing that it can break down lipids and proteins (i.e. casein) with the use of extracellular proteases and lipases^9,10^. Many *Brevibacterium* isolates also have the ability to modify sulfur-containing amino acids to produce volatile sulfur compounds which are important for flavor development^11–13^. *Brevibacterium* strains are thus often used as surface-ripening cultures in many different cheese types^8^. Understanding the functional potential of cheese bacteria is essential in the combined effort with cheese producers to shorten ripening times, reduce spoilage, better control cheese aroma, and increase food safety.

The taxonomy of the genus *Brevibacterium* is under reorganization with *Brevibacterium* genomes varying greatly in both size and functional content^7,14,15^. It should be noted that the genus *Brevibacterium* also contains opportunistic pathogens and other biotechnologically important species^16–19^. Cheese-associated subtypes of *Brevibacterium* can be found in several species of the genus, suggesting that adaptation to cheese environments might have been acquired independently through horizontal gene transfer (HGT) events^15,20^. Two recent studies have analyzed the genetic content of *Brevibacterium* focusing on their putative genetic functions in cheese production. The first provided evidence for the prevalence of HGT in cheese-associated *Actinobacteria* in general and the existence of highly conserved islands denoted iron uptake/siderophore transport island (RUSTI), which are presumed to be involved in iron uptake^20^. Iron acquisition capabilities are a key fitness advantage of cheese bacteria because of the scarcity of free iron in cheese and milk^20,21^. The second study analyzed 23 *Brevibacterium* isolate genomes, 12 of which were isolated from cheese, and found many putative genes involved in iron acquisition and bacteriocin production. Notably, they found a 96 kbp insertion element in a number of *Brevibacterium* genomes containing lanthipeptide bacteriocin genes, which they designated *Brevibacterium* Lanthipeptide Island (BreLI)^15^. These BreLI islands thus potentially provide a competitive advantage to their host strains against other bacteria.

A number of studies have shown that some *Brevibacterium* strains such as *B. aurantiacum* and *B. linens*, even if intentionally inoculated on surface-ripened cheeses, do not establish themselves during ripening^22–28^. Studies that assess abundance of *Brevibacterium* on the rind during cheese ripening are limited to a number of (semi-quantitative) 16S rRNA gene amplicon sequencing studies^3,28–30^; however, quantitative PCR approaches focusing on *Brevibacterium* are, to the best of our knowledge, confined to one study^31^. The latter study used reverse transcriptase qPCR to determine the abundance of *Brevibacterium* and other cheese rind bacteria and yeasts on French smear-ripened cheeses targeting ribosomal RNAs or different protein coding genes.

Histamine is a biogenic amine (BA) that is involved with the immune, cardiovascular, and gastrointestinal systems in mammals. However, in bacteria, its production is often associated with survival mechanisms responding to acidic environments^32–34^. Histamine can be produced in high concentration by bacteria during food fermentation^35^ with concentrations reaching up to 400 mg/kg depending on the type of cheese^36–38^. Consumption of histamine can lead to histamine intoxication; characterized by diarrhea, asthma, swelling, rash, hives and other allergic-like reaction conditions^34,39^. Long-ripened cheese is among the most commonly associated sources of dietary-acquired BAs, surpassed only by fish^37,40,41^. Histamine intoxication can be mitigated by preventing histamine formation or by degrading histamine in foods. One way to reduce histamine levels in cheese may thus be to add histamine-degrading bacteria to the cheeses during cheese production and ripening.

Vorarlberger Bergkäse (VB) is a long-ripened artisanal hard cheese derived from raw milk of cows grazing alpine pastures in the western region of Austria known as Vorarlberg. VB cheese wheels are regularly washed with brine or surface treated with dry salt. The ripening time may span from three to 18 months, during this time a rind consisting of bacteria and fungi will form on the cheeses. No external surface-ripening cultures are added. Our group has previously characterized the microbial community composition of VB using 16S and 18S rRNA gene cloning and sequencing^42^. Different *Brevibacterium* phylotypes have been characterized to be abundant on the rind and in production facilities of VB^6^. This study aimed to characterize the abundance of *Brevibacterium* on VB cheese rinds using qPCR and to analyze the contribution of *Brevibacterium* to cheese ripening based on draft genome sequences for three *Brevibacterium* isolates from VB: L261, S22, and S111. Their genetic potential in regards to cheese ripening was compared to other cheese-associated *Brevibacterium* strains. We hypothesize that the VB *Brevibacterium* isolates are habituated for the competitive environment of the cheese rind throughout the ripening time and contribute to the texture, color, and aroma of VB.

## Results and Discussion

### qPCR results

**Figure 1:**
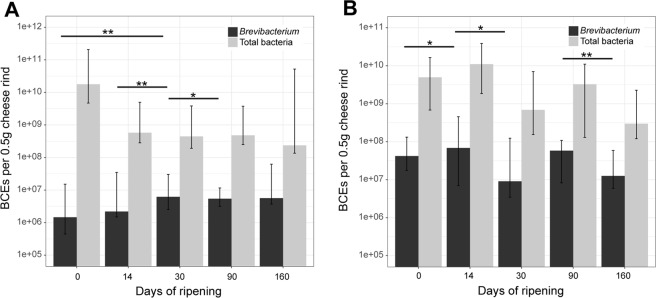
Abundance of *Brevibacterium* on cheese rinds during ripening of Vorarlberger Bergkäse in two different cheese production plants determined by qPCR. Bacterial cell equivalents (BCE) per 0.5 g cheese rind during ripening in two different cheese production facilities are shown. Graphs show median and interquartile ranges for the 20 samples from each plant [(**A**): plant A, and (**B**): plant B] for each analyzed day of ripening (0, 14, 30, 90 and 160 days). Statistically significant differences of *Brevibacterium* BCEs are highlighted by asterisks, with *indicating p < 0.05 and **indicating p < 0.01. Numerical BCE values are shown in Table S1, p-values are shown in Table S2.

The abundance of *Brevibacterium* on VB cheese rinds during ripening was determined through qPCR analysis. Overall, a higher abundance of *Brevibacterium* was found in plant B compared to plant A. At day 0, *Brevibacterium* was present in higher bacterial cell equivalents (BCEs) in plant B than in A (Median, 4.21e + 07 and 1.46e + 06, respectively) (Fig. 1, Tables S1 and S2). *Brevibacterium* BCEs from plant A were increasing significantly during the first 30 days (4.2 fold increase from 0 to 30 days, p < 0.01; 2.8 fold increase from 14 to 30 days, p < 0.01), and remained relatively constant during the rest of the ripening time. *Brevibacterium* BCEs in plant B changed significantly when comparing day 0 to 14 (1.6 fold increase, p < 0.05), day 14 to 30 (7.5 fold- decrease, P < 0.05) and day 90 to 160 (4.6 fold decrease, p < 0.01).

Our qPCR results show that the genus *Brevibacterium* is found in high BCEs on the surface of VB already at day 0 of processing and remained abundant throughout the observed ripening time of 160 days. Some studies have reported that *Brevibacterium* strains, even if inoculated on the rinds for ripening, do not establish themselves on cheese rinds^22–28^. This is in contrast to the notion that *Brevibacterium* are late-successional taxa on cheese surfaces^3,28^. Our previous study showed that *Brevibacterium* clones are abundant on VB cheese rinds and one of the *Brevibacterium* Operational Taxonomic Units (OTUs) increased significantly during cheese ripening^42^. It has been suggested that the abundance of *Brevibacterium* on cheese rinds is highly strain-dependent or dependent on the co-occurring cheese microbiota such as fungi. Growth of *Brevibacterium* on cheese rinds is stimulated by *Geotrichum candidum*^43^ and inhibited by *Penicillium*^3^. *Brevibacterium* is abundant on VB cheese rinds already at the first day of production; this suggests that *Brevibacterium* might originate in high numbers from the raw milk used for production of VB. In line with this, *Brevibacterium* have often been found in raw milk^44^. Raw milk-associated *Brevibacterium* seem to be the most likely possible source of transmission as a recent study from our group indicated that *Brevibacterium* is the second most abundant clone from both VB rinds and ripening cellar environments^6^. A possible inoculation of VB rinds with *Brevibacterium* in such high numbers as reported here from the production environment in such short time (i.e. at the day of production) seems to be unlikely. These results do not prove a source of transmission, the origin of the cheese rind *Brevibacterium* strains needs to be verified experimentally in future studies.

### Isolation of bacteria from VB rinds

**Figure 2:**
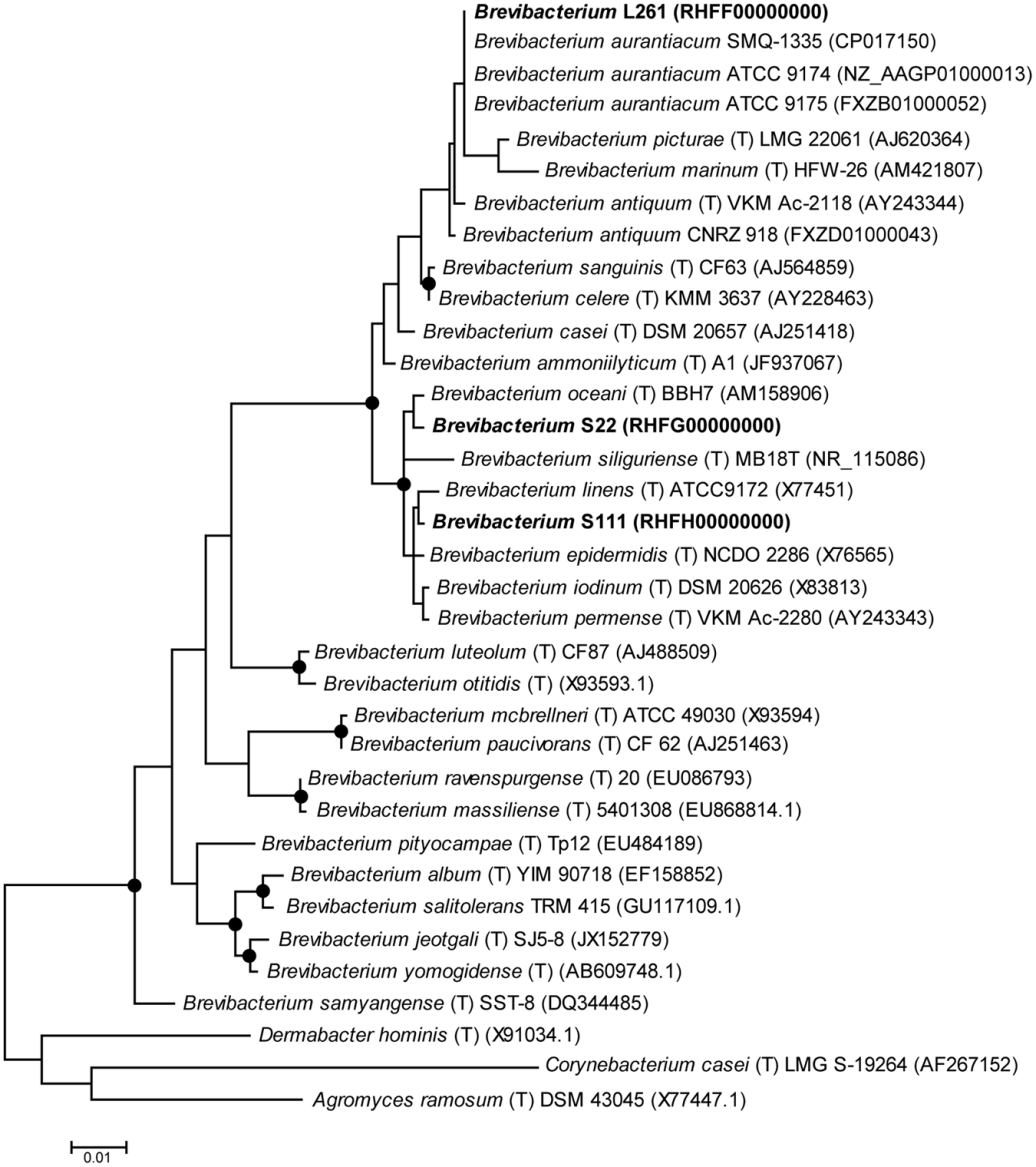
Phylogenetic relationships of *Brevibacterium* strains based on 16S rRNA gene sequences. The evolutionary history was inferred by using the Maximum Likelihood method based on the Tamura-Nei model. The tree with the highest log likelihood (−4232.79) is shown. The tree is drawn to scale, with branch lengths measured in the number of substitutions per site. The analysis involved 35 nucleotide sequences. All positions containing gaps and missing data were eliminated. There were a total of 1127 positions in the final dataset. Evolutionary analyses were conducted in MEGA7^85^. Isolates obtained in this study are highlighted in bold. Type strains are indicated by “(T)”, GenBank accession numbers are shown in parentheses. Black dots indicate Maximum Likelihood, Neighbor-Joining and Maximum Parsimony bootstrap values higher than 85 (1000x resampling).

To obtain cheese rind bacteria isolates for functional characterization such as genome sequencing, we performed a cultivation approach on VB cheese rinds yielding a total number of 143 isolates, see^45^ for details. Out of these isolates, 10 were identified as *Brevibacterium*. Based on a 99% 16S rRNA gene sequence similarity threshold, these isolates belonged to three OTUs (data not shown). From each of these OTUs, one strain was selected for whole genome sequencing. *Brevibacterium* S22 and L261 were isolated from one month old cheese rinds, *Brevibacterium* S111 was isolated from six months old cheese samples. 16S rRNA gene-based phylogenetic analysis of the three new isolates from this study with other *Brevibacterium* strains revealed that all three strains clearly clustered within the genus *Brevibacterium* (Fig. 2). *Brevibacterium* L261 clustered together with *B. aurantiacum*, whereas S22 and S111 clustered together with the *B. linens*/*B. siliguriense*/*B. iodiunum* group recently postulated by^15^. *Brevibacterium* L261 shows yellow-orange pigmentation and *Brevibacterium* S111 showed beige pigmentation, whereas *Brevibacterium* S22 colonies are white.

### Similarity of VB cheese rind isolates to VB cheese rind and production environment clones

The near full-length 16S rRNA gene sequences of S22, S111 and L261, which were isolated in 2014, were compared to 16S rRNA gene clone sequences from our previous studies that analyzed VB rinds and production environments sampled in 2012 from the same cheese production plants using BLASTn^6,42^. L261 showed 98.8% identity to OTU2 from^42^ and 99.6% identity OTU3 from^6^ and S111 showed 99.8% identity to OTU19 from^42^. S22 showed less than 98% identity to *Brevibacterium* OTUs from^42^ and 99.8% identity to OTU18 from^6^. These results suggest that different *Brevibacterium* strains or species can be found in the VB cheese rind communities and also in the VB production environment for over a time period of several years, although it should be noted that, based on 16S rRNA similarity alone, a differentiation of strains is not possible; for this, approaches with higher taxonomic resolution such as Pulsed-Field Gel Electrophoresis (PFGE) or Multi Locus Sequence Typing (MLST) would be needed.

### Genome sequencing and analysis of *Brevibacterium* from VB rinds

Illumina MiSeq sequencing and assembly yielded between 70 and 100 contigs for each strain with an average coverage of 160× for S22, 255× for S111, and 275× for L261. *Brevibacterium* L261 had an assembly size of 4.48 Mbp within 100 contigs, the assembly size for L261 is larger than those of other *B. aurantiacum* isolates (Table 1), which is consistent with the described variability of genome sizes in the genus *Brevibacterium*^15^. The genomic GC content of L261 (62.8%) was highly similar to other *B. aurantiacum* isolates (62.6 to 62.8%). L261 average nucleotide identity (ANI) and 16S rRNA gene sequence similarity were above the threshold of species demarcations (ANI > 95%, 16S rRNA gene > 99%)^46,47^ to sequenced *B. aurantiacum* genomes (*B. aurantiacum* SMQ-1335, *B. aurantiacum* ATCC 9174, and *B. aurantiacum* ATCC 9175), suggesting that L261 belongs to the species *B. aurantiacum*. Also, Pham *et al*.^15^ considered *Brevibacterium* genomes belong to the same species with 16S rRNA gene similarity >98% and ANI > 95%. Similarly, also tetranucleotide correlation analyses revealed that these four *B. aurantiacum* genomes had tetra correlation values higher than 0.999, which is also indicative of belonging to the same species (Table S3). *B. linens* has been split into four different species: *B. linens*, *B. aurantiacum*, *B. antiquum* and *B. permense*^14^ which was confirmed by recent genome-based phylogenetic analyses^15^. *Brevibacterium* S22 and S111 reads were assembled into 72 and 70 contigs with assembly sizes of 4.51 and 4.04 Mbp, respectively. ANI (<86%) and 16S rRNA gene similarities (<98.6%) of S22 and S111 were below the threshold of species demarcation when compared to other *Brevibacterium* type strains analyzed, thus S22 and S111 probably represent novel species in the genus *Brevibacterium*. Similarly, also the tetranucelotide correlation analyses values for S22 and S111 were below the cutoff (<0.996) for species demarcation (Table S3). Unambiguous and formal description of S22 and S111 as novel species of the genus *Brevibacterium* would require additional experimental work which was outside the scope of this study.

**Table 1:** Overview on *Brevibacterium* strains included in this study.

	*B. aurantiacum* L261	*Brevibacterium* S111	*Brevibacterium* S22	*B. aurantiacum* SMQ-1335	*B. aurantiacum* ATCC 9174	*B. aurantiacum* ATCC 9175	*B. antiquum* CNRZ 918	*B. casei* CIP 102111	*B. linens* ATCC 9172	*B. iodinum* ATCC 49514
Assembly size (Mbp)	4.481	4.043	4.507	4.209	4.366	4.147	3.748	3.840	3.959	3.535
Reference	^42^	^42^	^42^	^88^	Bioproject PRJNA405	^15^	^15^	^15^	^15^	^15^
Source	VB, Austria	VB, Austria	VB, Austria	Cheese	Romadour cheese, Germany	Camembert cheese	Beaufort cheese, France	Cheddar cheese	Harzer cheese, Germany	Milk
No of contigs	100	70	72	1	76	70	49	24	80	65
16S rRNA similarity to L261		96.7%	96.4%	99.9%	99.3%	99.7%	99.0%	97.3%	97.0%	96.3%
16S rRNA similarity to S111	96.7%		97.8%	96.7%	96.4%	96.7%	96.8%	97.5%	98.6%	98.4%
16S rRNA similarity to S22	96.4%	97.8%		96.7%	96.7%	96.3%	96.9%	97.2%	98.1%	97.4%
ANI* to L261 (%) [coverage]		78.89 [55.25]	78.82 [55.43]	96.02 [76.23]	95.73 [78.68]	96.12 [75.99]	86.2 [62.11]	77.21 [48.56]	78.97 [54.61]	78.62 [50.94]
ANI* to S111 (%) [coverage]	79.26 [60.75]		84.04 [68.39]	78.6 [57.57]	78.71 [59.2]	78.59 [58.07]	77.9 [55.17]	77.77 [51.23]	83.82 [63.9]	83.47 [60.47]
ANI* to S22 (%) [coverage]	78.69 [55.13]	83.91 [61.88]		78.18 [52.71]	78.54 [54.06]	78.38 [53.39]	77.86 [48.87]	77.38 [45.81]	86.44 [60.41]	86.65 [58.53]
GC content	62.8%	65.0%	64.1%	62.6%	62.8%	62.7%	62.7%	68.0%	64.7%	64.5%

*ANI was calculated with the Blast algorithm using the JSpeciesWS Webserver.

### Functional potential for cheese ripening

To characterize the functional potential of *Brevibacterium* isolates for cheese production, we analyzed the annotated genomes for enzymes characterized in previous studies to be important for cheese ripening. Sulfur-containing amino acids have been described as important precursors for volatile sulfur compounds responsible for flavor of cheeses^13,48^. Genomes of *Brevibacterium* L261, S111, and S22 harbor three copies of a putative methionine aminopeptidase (EC 3.4.11.18) and encode one homolog of the methionine gamma-lyase (EC 4.4.1.11, MGL), which shows high amino acid identity (94%, 80%, and 78%, respectively) to the characterized MGLs from *B. aurantiacum* ATCC 9175 and ATCC 9174^11,49^. The locus_tags for the homologs of the cheese enzymes discussed in this section in *Brevibacterium* L261, S111, and S22 are listed in Table S4. MGLs can produce methanethiol from methionine, which in turn is converted to various volatile sulfur containing compounds important for aroma of the cheese^13,48^. Proline and glutamate are found in high abundance in casein^50–52^. Isolates S22, S111, and L261 can metabolize proline through one proline iminopeptidase (EC 3.4.11.5) and one Xaa-Pro aminopeptidase (EC 3.4.11.9). In addition, L261, S111, and S22 harbor homologs with high amino acid identity (≥68%) to the cell wall-associated protease characterized from *B. aurantiacum* ATCC 9174^53^. Furthermore, different aminopeptidases have been described in *Brevibacterium* to be important for cheese ripening^9,54,55^. L261, S22 and S111 contained homologs with high similarity (92 to 100% amino acid identity) to the N-terminal sequence of aminopeptidase II identified in *B. linens* SR3 by^55^. L261, S22 and S111 also contain homologs with high similarity (63% amino acid identity) to the N-terminal sequence of an aminopeptidase characterized in *B. aurantiacum* ATCC 9174 by^56^. Glutamate can be metabolized by employing three copies of glutamate dehydrogenase (EC 1.4.1.2). Aminotransferases are important in the transformation of amino acids into aroma compounds during cheese production^12,48,57^. Isolates S22, S111, and L261 harbor both putative aromatic and branched-chain aminotransferases (EC 2.6.1.57 and 2.6.1.42). Lipolytic enzymes such as esterases are important in cheese ripening and esterase activity has been described in *Brevibacterium* before^9,10^. Rattray and Fox^58^ have purified an intracellular esterase from *B. aurantiacum* ATCC 9174; L261, S22, and S111 contain homologs to the N-terminal sequence of the purified esterase sharing 84 to 94% amino acid identity. The L261, S22, S111, and all other genomes analyzed, harbor a cluster of genes possibly involved in phenylacetate degradation. Phenylacetate has been described to be responsible for off-flavor in Cheddar^59^, but also key for flavor production in Swiss-type cheeses^60^.

### Protease and lipase activities of *Brevibacterium* isolates

The *Brevibacterium* isolates S22, S111, and L261 were analyzed for their protease and lipase activity by qualitatively scoring halo formation on skim milk and Spirit Blue agars, respectively. Protease and lipase activity was observed in all three isolates. By comparing the relative size of halos between strains it was determined that S111 showed the highest activity when assessed for proteolytic and lipolytic activity after 14 days; after 21 days, the halo sizes were similar for all three strains (Table 2).

**Table 2:** Proteolytic and lipolytic activity of *Brevibacterium* isolates S22, L261, and S111.

Proteolytic activity (skim milk agar plates)	Halo size day 7	Halo size day 14	Halo size day 21
*Brevibacterium* S22	small	small	large
*Brevibacterium* S111	small	large	large
*B. aurantiacum* L261	small	medium	large
**Lipolytic activity** (spirit blue agar plates)			
*Brevibacterium* S22	medium	medium	large
*Brevibacterium* S111	small	large	large
*B. aurantiacum* L261	small	medium	large

### Differences between L261, S22, and S111

While many described and identified genes important for cheese ripening are conserved between different *Brevibacterium* strains, we also identified a number of different features between the three VB isolates. Isolate S22 (but not L261 and S111) contains an *ureABCDFG* gene cluster for degradation of urea; some *Brevibacterium* strains have been described to be able to degrade urea^14^. The presence of a urease gene cluster could enable *Brevibacterium* to degrade urea to ammonia and thereby increase the pH on the cheese rind. Homologs of this cluster were also identified in the cheese isolates *B. linens* ATCC 9172 and *B. casei* CIP 102111. The growth of *Brevibacterium* on cheese is stimulated by vitamin production of yeasts and fungi^9^, we found that strain L261 encodes all genes necessary for biotin production (*bioABCDFH*), while S22 and S111 encode only an incomplete biotin biosynthesis pathway. All strains analyzed in this study also encode a BioMNY putative biotin transporter. The presence of a complete biotin synthesis pathway might provide advantages to L261 during growth on cheese rinds.

### *Brevibacterium* S22 encodes a novel 22 kb plasmid

A putative plasmid contig was identified in *Brevibacterium* S22, it has a size of 22.4 kbp, a GC content of 64.4% and encodes 25 predicted genes, two of them show high amino acid identity to the RepA and RepB proteins (between 74 and 63%, respectively) from the pLIM, pRBL1 and pBLA8 plasmids from *B. linens*^61–63^. The coverage of the plasmid contig was 1763×, which is 11-fold higher than the average coverage of the chromosomal contigs (160×) suggesting that the S22 plasmid is a medium-copy number plasmid. Agarose gel electrophoresis and restriction enzyme digest confirmed the size and presence of a plasmid, which we named pBS22 (Fig. S1). PCR assays targeting the ends of the plasmid contig revealed PCR products of approximately 500 bp and demonstrated that pBS22 is a circular plasmid (data not shown). Homologs to a hydroxylmethyl pyrimidine (HMP)/thiamine ABC transporter described in *Bacillus subtilis* (YkoCDE)^64^, an iron import system (IrtAB) functionally characterized in *Mycobacterium tuberculosis*^65,66^, and a multi-drug resistance pump (Stp), shown to increase tolerance to spectinomycin and tetracycline characterized also in *Mycobacterium tuberculosis*^67^, were identified on pBS22 (Fig. S2). Some of the other predicted proteins on the plasmid were annotated as transcriptional regulators or transposases, and a few could not be assigned with a putative function based on sequence analysis. Most of the plasmid genes show highest similarity to bacteria other than *Brevibacterium*. The putative IrtAB iron transporters show highest amino acid identity (73 and 77%) to the cheese isolate *Gulosibacter sp* 10^68^ and *Agrococcus casei* LMG22410^68^ (62 and 67% amino acid identity); the amino acid identity to *Brevibacterium* homologs is below 47%. The putative YkoCDE transporters show also highest similarity to homologs in *Gulosibacter sp* 10 (72 to 78% amino acid identity) and various non cheese-derived *Actinobacteria* (63% to 67% amino acid identity); the amino acid identity to *Brevibacterium* homologs is below 43%. Plasmids such as pBL33 (7.5 kb), pLIM (7.6 kb), pBL100 (7.7 kb), or pRBL1 (8 kb) have been identified in some *Brevibacterium* isolates, but so far, the function of these plasmids remains unknown^9,61–63,69,70^. These plasmids belong to the family of theta-replicating ColE-related plasmids^61–63^. Phylogenetic analyses of plasmid RepA protein sequences revealed that the pBS22 RepA protein clustered consistently together, but more distant and more deeply with other RepA proteins from the genus *Brevibacterium* (Fig. S3). pBS22 lacks homologs of the ORFIII proteins found in many *Brevibacterium* plasmids (Table S5), but encodes putative MobAC plasmid mobilization proteins which do not show similarity to other *Brevibacterium* proteins. Recently, a 89 kb linear plasmid, pAP13, was identified in a *Brevibacterium* isolate from feces^71,72^ but also for this plasmid, no putative function is currently known. pAP13 shows no similarity to pBS22 determined by BlastP analyses. The identification of pBS22, which is putatively involved in iron and vitamin uptake and might thus provide an adaptive advantage on cheese rinds, is thus the first description of a non-cryptic plasmid in *Brevibacterium*. An increased knowledge about the distribution and potential function of *Brevibacterium* plasmids as performed in this study is a prerequisite for identifying plasmids and – in the long term - to be able to develop a genetic system for *Brevibacterium*. This is of particular relevance given the importance of *Brevibacterium* as cheese ripening strains and that transformation of *Brevibacterium* has been described only in a few strains and transformation efficiency is highly strain dependent^62,73^.

### Possible plasmid content in other *Brevibacterium* strains

When using the pLIM RepAB and the replication-associated ORFIII proteins as query for BlastP searches against other *Brevibacterium* genomes, we identified highly similar homologs with more than 95%, 68%, and 89% amino acid identity (RepA, RepB, ORFIII, respectively) in a number of *Brevibacterium* strains including *B. aurantiacum* ATCC 9174 (Table S5). Interestingly, the homologs in *B. aurantiacum* ATCC 9174 are located on one contig with a size of 8.7 kb, which is highly similar to the size of pBL33 which has been purified from *B. aurantiacum* ATCC 9174 and analyzed by restriction enzyme analyses to be 7.5 kb previously^69^. It is thus likely that this *B. aurantiacum* ATCC 9174 contig represents the cryptic plasmid pBL33. Based on the similarity of RepAB and ORFIII proteins, and the clustering of RepA proteins (Fig. S3) and the presence of these homologs on contigs with sizes ranging from 5.2 to 9 kb, we speculate that small cryptic plasmids with replication proteins highly similar to pLIM, pRBL1, and pBLA8 are found in a higher number of *Brevibacterium* strains than previously anticipated. It should be noted that these sequence analyses do not prove whether these contigs do actually represent complete or partial plasmids.

### Comparative genomic analysis of previously described *Brevibacterium* genomic islands and bacteriocin loci

Iron is a limiting resource for cheese microorganisms^5,21^. The recently described RUSTI and BreLI islands^15,20^ are absent in *Brevibacterium* isolates S22, S111, and L261. However, homologs of smaller previously described bacteriocin gene clusters were found: L261, S22 and S111 encode a putative Linocin-M18 bacteriocin. Linocin-M18 was isolated from *Brevibacterium linens* M18 and inhibits the growth of many gram-positive bacteria, including species of the genus *Listeria*^74^ and is found in many *Brevibacterium* strains and coryneform bacteria^15^. L261 encodes homologs of a lactococcin 972-related bacteriocin and of a linear azol(in)e-containing peptide gene cluster recently identified by^15^.

### Histamine metabolism

Sequence analysis of isolates S22, S111, and L261 revealed homologs to a recently described histamine catabolism pathway in *Pseudomonas putida*^75^ (Table 3, Fig. S4). Isolates S22, S111, and L261 have homologs with high similarity (amino acid identity ≥ 40%) to HinADFGHIL proteins and lower, but still high, similarity (amino acid identity 32 to 36%) to HinC and HinK. In addition, we found highly similar homologs (≥52% amino acid identity) of a functionally characterized histamine oxidase (E.C. 1.4.3.22) from *Arthrobacter globiformis*^76^ in S22, L261, and S111. This histamine oxidase could fulfil the function of HinC, for which only a homolog with lower similarity has been found using the *Pseudomonas putida* HinC as query. HinC from *Pseudomonas putida* is a histamine-deaminase/histamine-pyruvate aminotransferase that catalyzes the oxidation of histamine to imidazole acetaldehyde. Similar to HinC, histamine oxidase also catalyzes the conversion of histamine to imidazole acetaldehyde. In the *Brevibacterium* strains S22, L261 and S111, homologs of both HinC and the *Arthrobacter* histamine oxidase might have the potential to perform the initial step of histamine degradation. Some *Pseudomonas putida* histamine genes homologs were absent in the *Brevibacterium* strains analyzed here. HinE is an aldehyde dehydrogenase that complements HinD in the oxidation of imidazole acetaldehyde to imidazole-4-acetate. Histamine degradation was not hindered in *hinE* deletion mutants^75^, thus it is a nonessential gene for histamine degradation. We found no homologs of HinB and HinJ, which are transcriptional regulators; the absence of clear homologs of transcriptional regulators might be explained by different transcriptional regulation of histamine degradation genes between *Pseudomonas putida* and *Brevibacterium*, which belong to different phyla.

**Table 3:** Homologs of *Pseudomonas putida* histamine catabolism enzymes in *Brevibacterium* L261, S111, S22.

*P. putida* protein (GenBank accession number)	*Brevibacterium* L261 Amino acid identity (%), [coverage %], NCBI GenBank locus_tag	*Brevibacterium* S111 Amino acid identity (%), [coverage %], NCBI GenBank locus_tag	*Brevibacterium* S22 Amino acid identity (%), [coverage %], NCBI GenBank locus_tag
HinA permease (AWA45220)	50 [98], EB834_11790	51 [98], EB836_13825	50 [98], EB835_06785
HinD EC 1.2.1.3 aldehyde dehydrogenase (AWA45224)	50 [98], EB834_10860	50 [99], EB836_11485	50 [99], EB835_13590
HinF EC 1.14.13.5 FAD-monooxygenase (AWA45229)	63 [92], EB834_10890	60 [94], EB836_11515	61 [92], EB835_13560
HinG EC 3.5.1.8 amidase (AWA45228)	43 [98], EB834_09805	42 [98], EB836_03650	40 [97], EB835_02985
HinH amidase (AWA45227)	48 [100], EB834_10875	50 [100], EB836_11500	48 [100], EB835_13575
HinI aspartate ammonia-lyase EC 4.3.1.1 (AWA45233)	56 [98], EB834_10895	56 [96], EB836_11520	54 [97], EB835_13555
HinL enamine deaminase (AWA45230)	65 [98], EB834_10880	61 [99], EB836_11505	64 [98], EB835_13570

The other *Brevibacterium* genomes analyzed in this study encode homologs of only some of the genes in the *Pseudomonas putida* histamine degradation pathway described by^75^ (Table S6, Fig. S4). Based on this, we speculate that the histamine degradation potential may be absent in the other *Brevibacterium* genomes analyzed here. It should be noted that some of the other *Brevibacterium* strains analyzed here contain homologs of the histamine oxidase from *Arthrobacter globiformis*, they could therefore perform the initial step of histamine degradation and might use other, yet unknown, pathways for complete histamine degradation. However, proof of the presence or absence of histamine degradation potential would need to be verified experimentally in those strains in future studies.

**Figure 3:**
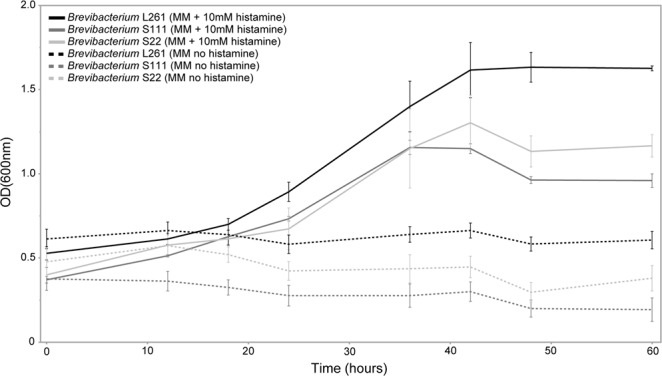
Growth of *Brevibacterium* strains S22, S111, and L261 in minimal medium (MM) with or without 10 mM histamine as sole carbon source. Growth was determined using optical density measurements at 600 nm (OD_600_). Values represent mean values ± SEM.

To provide experimental evidence that *Brevibacterium* L261, S111, and S22 can degrade histamine, we performed growth experiments in minimal medium (MM) with or without 10 mM histamine as the sole carbon source. The growth curves revealed that all three strains are capable to utilize histamine and grow in MM supplemented with 10 mM histamine (Fig. 3). Growth occurred to a maximal optical density (OD) of 1.1 to 1.6 (depending on the strain) after reaching those maximal ODs, the cultures switched to stationary phase. No growth was observed for the strains cultured in MM without added histamine. These results show that *Brevibacterium* L261, S111, and S22 can utilize histamine. However, to determine if the genes in the putative histamine degradation pathway are responsible for histamine degradation, needs to be verified in future experiments. Our results are in line with previous reports that have described histamine degradation for *Brevibacterium* strains^77,78^; however, the molecular pathway responsible for histamine degradation in *Brevibacterium* has not been identified until now. Here, we provide evidence for histamine degradation and the presence of a putative histamine degradation pathway in *Brevibacterium* strains L261, S22, and S111 which could enable them to reduce levels of histamine in hard cheeses.

Furthermore, all genomes in this study were then analyzed for their potential ability to produce histamine by a histidine decarboxylase (E.C. 4.1.1.22) similar to other cheese bacteria^37^. No homologs of histidine decarboxylases were identified, suggesting that all strains are incapable of histamine synthesis.

## Conclusion

Here we report the draft genome sequences of *Brevibacterium* isolates from Austrian hard cheese rinds. One of the isolates (L261) belonged to *B. aurantiacum*, and two of the isolates (S22 and S111) most likely represent novel *Brevibacterium* species. Our qPCR results show that *Brevibacterium* strains are abundant members of the VB cheese rind communities throughout 160 days of ripening. The genomes of *Brevibacterium* strains S22 and S111 provide further evidence for the diversity of the genus *Brevibacterium* in general and of cheese-associated *Brevibacterium* in particular. Our results show that many important enzymes for cheese ripening are conserved among cheese-associated *Brevibacterium*. *Brevibacterium* S22 harbors a plasmid which might provide adaptation advantages on cheese rinds by encoded putative iron and thiamine import proteins. We reveal evidence for the presence of a potential metabolic pathway responsible for histamine degradation which is found in cheese-associated *Brevibacterium* strains L261, S22, and S111. Growth experiments revealed that these isolates are able to degrade histamine which underscores the importance of *Brevibacterium* strains as cheese ripening cultures.

## Methods

This study used the same samples for qPCR and cultivation as described recently in detail in^45^.

### Cheese rind sampling

Cheese rind samples were obtained from VB cheese wheels in ripening cellars of two different cheese production operations (abbreviated A and B) located in Vorarlberg, Austria. Samples were taken in March 2014 from 20 cheese wheel rinds directly after production (day 0) and at 14, 30, 90 and 160 days of ripening time.

### Isolation and identification of cheese rind bacteria

The cultivation of cheese rind bacteria followed the procedures described in^45^. Briefly, two grams of each sample were homogenized in 20 mL of sterile Ringer solution using a Stomacher 400 blender. Aliquots of 100 µL were diluted in sterile Ringer Solution and plated on modified ATCC medium 1097 [casamino acids 7.5 g^−L^; proteose peptone 5.0 g^−L^; yeast extract 1.0 g^−L^, sodium citrate 3.0 g^−L^, MgSO_4_ × 7H_2_O 20.0 g^−L^, K_2_HPO_4_ 0.5 g^−L^, Fe(NH_4_)_2_(SO_4_)_2_ × 6H_2_O, vancomycin 5 mg^−L^, crystal violet 5 mg^−L^, 8% (wt/vol) NaCl] under aerobic conditions at 37 °C. The main focus of our cultivation approach was to enrich for Gram-negative bacteria^45^; thus, vancomycin and crystal violet were used as described in^79^. Nevertheless, also many Gram-positive isolates were retrieved in spite of the vancomycin and crystal violet added to the original growth media (data not shown). DNA was isolated with the NucleoSpin Tissue DNA Extraction Kit (Macherey-Nagel) using the manufacturer’s recommended specifications. The extracted DNA was used for 16S rRNA gene PCR amplification using primers 27 F (5′-AGA GTT TGA TCM TGG CTC AG-3′) and 1492 R (5′-GGY TAC CTT GTT ACG ACT T-3′) applying the following amplification conditions: initial denaturation at 95 °C for 5 minutes, followed by 30 cycles at 94 °C for 40 seconds, at 52 °C for 40 seconds, at 72 °C for 60 seconds and final extension at 72 °C for 7 minutes. Purification of PCR amplicons was done using the GeneJet PCR Purification Kit (Thermo Fisher Scientific) and sequenced using a Sanger platform to produce near full-coverage of the 16S rRNA gene sequences.

### Genome sequencing, assembly, and analysis of *Brevibacterium* isolates

DNA was isolated by employing the Qiagen Genomic-tip columns (20/G) using the manufacturer’s recommended specifications. An Illumina MiSeq platform (Microsynth, Balgach, Switzerland) was used for genome sequencing with paired-end-sequencing chemistry and 300 base pair (bp) read length. One Illumina Nextera XT library with a 1 kbp insert size was prepared for each genome. The reads for each strain were assembled with SPAdes^80^. The draft genome sequences of the *Brevibacterium* isolates were annotated and analyzed using PATRIC, www.patricbrc.org ^81^. Annotations of genes of interest were confirmed using NCBI BLASTp, Uniprot^82^, and Pfam webservers^83^. The proteomes of isolates L261, S22, and S111 were compared with other *Brevibacterium* strains using PATRIC Blastp and proteome comparison tools. The average nucleotide identity (ANI) and tetranucleotide correlation analyses between isolates were determined using the JSpeciesWS online server^84^. Phylogenetic relationships of *Brevibacterium* strains based on 16S rRNA were calculated with MEGA 7 using maximum likelihood, neighbor joining and maximum parsimony with 1000× bootstrapping^85^. For the maximum likelihood tree shown in Fig. 2, initial tree(s) for the heuristic search were obtained automatically by applying Neighbor-Join and BioNJ algorithms to a matrix of pairwise distances estimated using the Maximum Composite Likelihood (MCL) approach and then selecting the topology with superior log likelihood value.

### Accession numbers

This Whole Genome Shotgun project was deposited at DDBJ/ENA/GenBank under the accession XXXX00000000. The version described in this paper is version XXXX01000000. The genomes of L261, S22, and S111 have been deposited under the accession numbers RHFF00000000, RHFG00000000, and RHFH00000000 respectively. Raw reads were submitted to the NCBI Sequence Read Archive (SRA) with the Bioproject accession number PRJNA498327.

### qPCR to determine the abundance of Brevibacterium on cheese rinds

#### DNA extraction

Ten grams of cheese rind samples were homogenized in 30 mL of sterile Ringer Solution and centrifuged to produce a 250 mg pellet^45^. Genomic DNA was isolated from the pellets by utilizing the PowerSoil DNA Isolation kit (MoBio) following the producers guidelines. Aliquots (250 µL) were pooled in duplicate and DNA concentrations were determined with a Qubit^®^ 2.0 Fluorometer (Thermo Fisher Scientific).

### qPCR analysis of 16S rRNA genes

To assess differences in abundance between cellars from two dairy production plants and alterations in the absolute abundance in respect to ripening time, the amount of total bacteria and of *Brevibacterium* were quantified using qPCR. The data for 16S rRNA gene PCR analysis of total bacterial numbers in VB cheese rind samples was taken from Schmitz-Esser, 2018^45^. We used previously designed primers to amplify a 125 bp target region of the 16S rRNA genes of the genus *Brevibacterium*, 16S_Ba838-856 (F) (5′-GTA CGG TCG CAA GGC TAA A-3′) and 16S_Ba921-904 (R) (5′-TCC AGA ACG GTC TGG TGT-3′)^31^.

Negative controls without template were included in each qPCR reaction. The specificity of the amplicons was verified by DNA sequencing of the PCR products (LGC Genomics, Berlin, Germany), showing 99 to 100% identity to the 16S rRNA gene sequences of their respective target organisms. qPCR conditions and primers were optimized to obtain high PCR amplification efficiency of the target included in the qPCR assay as described in^86^ (Table S7). Each optimized qPCR reaction was run in duplicate with a final volume of 25 μL, using MicroAmp 0.2 mL optical tubes sealed with MicroAmp optical 8-cap strips (Applied Biosystems). Single amplification reactions for *Brevibacterium* qPCRs consisted of 11.95 μL diethylpyrocarbonate (DEPC)-treated water, 2.5 μL10× buffer, 1.75 μL 3.5 mM MgCl_2_,0.75 μL 300 nM of each primer, 1 μL 3.3 mM SYTO9 (Invitrogen), 1 μL 200 mM of each dNTP, 0.3 μL 1.5 U of Platinum® Taq DNA polymerase (Thermo Fisher Scientific) and 5 μL template (genomic DNA). The quantification of DNA was performed in Mx3000P™ qPCR system (Stratagene) (software v.4.10) after initial denaturation at 94 °C for two min, followed by 45 cycles of 94 °C for 30 s, 60 °C for one min. To determine the specificity of the amplifications, dissociation curves after each reaction were recorded and carried out at 95 °C for one min, followed by complete annealing at 50 °C for 30 s, and a gradual increasing temperature up to 95 °C. Post-run melting curves were checked for the presence of multiple peaks due to primer-dimers or nonspecific amplification. Additionally, to check for the presence of non-specific products and size of the amplicons, aliquots of qPCR products were analyzed by agarose gel electrophoresis.

DNA isolated from *Brevibacterium* L261 was used as the DNA qPCR standard to determine the absolute abundance of *Brevibacterium*, expressed as the bacterial cell equivalents (BCE) per 0.5 g cheese rind. The DNA concentration was determined fluorimetrically using a Qubit® 2.0 Fluorometer. When based on the mean molecular weight of 23 *Brevibacterium* genomes^15^ and three sequenced isolates described in this study (3,879,418 bp), 1 ng DNA equals 2.39 × 10^5^ copies of the entire genomes. The 16S rRNA gene copy numbers (mean: three copies for *Brevibacterium*^15^) were taken into account when extrapolating BCE per 0.5 g rind cheese from the qPCR.

The qPCR data (BCE per 0.5 g cheese rind) were analysed and compared using R (version 3.2.5, psych package 1.6.12). The dataset was divided into 10 different subsets based on the location (cheese production facility A, B) and days of ripening (0, 14, 30, 90 and 160). Because the Shapiro-Wilk test did show normal distribution for only one of the 10 subsets, all subsets were described by Median and interquartile ranges (IQR). Furthermore, the Wilcoxon Signed-Rank test was used to determine statistical differences (at a significance level ≤ 0.05) between subsets with the same location based on days of ripening. A p-value ≤ 0.05 was considered statistically significant. Due to the fact, that no relationship between the two cheese facilities (A and B) were found (Kendall’s Rank correlation Tau-B), observed qPCR data from two facilities were analysed separately.

### Purification, and analysis of the *Brevibacterium* S22 plasmid

A putative plasmid was identified in *Brevibacterium* S22 through sequence analysis and was designated pBS22. To confirm the presence of this plasmid, *Brevibacterium* S22 was cultivated overnight in Brain Heart Infusion broth (BD Biosciences) with 3% (wt/vol) added NaCl at 37 °C and shaking at 200 rpm. Plasmid DNA was purified using the GeneJET Plasmid Minprep Kit (Thermo Scientific). Plasmid DNA was linearized with the restriction enzyme HindIII (Thermo Scientific) and loaded on an agarose gel. Primers pBS22Fwd (5′-TCA GTG AGC AAC GTG AGG-3′) and pBS22Rev (5′-TAT GCC AGA CAT GTC GGG-3′) were used to elucidate if pBS22 is a circular plasmid. PCR conditions were as follows: initial denaturation at 94 °C for 3 minutes, followed by 35 cycles at 94 °C for 30 seconds, at 52 °C for 30 seconds, at 72 °C for 60 seconds and a final extension at 72 °C for 7 minutes. The PCR amplicons were purified using the PureLink^TM^ Quick PCR Purification Kit (Invitrogen by Thermo Fisher Scientific) and sequenced using a Sanger platform to complete the plasmid sequence.

### Proteolysis and lipolysis screening

*Brevibacterium* isolates L261, S22, and S111 were screened qualitatively for lipolytic and proteolytic properties. Cultures were grown overnight in Brain Heart Infusion broth (BD Biosciences) with 3% (wt/vol) added NaCl and incubated at 30 °C and shaking at 210 rpm. Cultures were diluted to an OD_600_ of 0.2. 10 µL of each diluted culture was transferred on the center of a skim milk agar plate [5% (wt/vol) skim milk (BD Biosciences), 1% (wt/vol) yeast extract (BD Biosciences), and 2% (wt/vol) agar (Thermo Fisher Scientific)] and on a Spirit Blue Agar plate (BD Biosciences) [2% (wt/vol) agar, 1% (wt/vol) tryptone, 0.5% (wt/vol) of yeast extract, 0.015% (wt/vol) spirit blue, 3% (vol/vol) lipase reagent] in duplicate to assess proteolytic and lipolytic activities following recently published procedures^10^. Plates were incubated at 20 °C. Observations occurred once every seven days for a total of 21 days. Presence and size of clear haloes on the agar plates indicate proteolytic and lipolytic activity.

### Growth of *Brevibacterium* in minimal medium with histamine

To test if the *Brevibacterium* isolates S22, S111, and L261 can utilize histamine, we determined growth curves in a defined MM containing histamine as sole carbon source^75,87^. The MM consisted of (gL^−1^): KH_2_PO_4_ (13.6), (NH_4_)_2_SO_4_ (2.0), MgSO_4_*7H_2_O (0.25), FeSO_4_*7H_2_O (0.0005), biotin (0.0003), thiamine hydrochloride (0.0012), and calcium panthotenate (0.00015). The pH was adjusted to 8. This medium was used with or without addition of 10 mM histamine as carbon source. Test tubes containing 5 ml MM were inoculated with the one loop (approx. 10 µl) bacterial colony grown on marine broth –tryptic soy agar plates consisting of (gL^−1^): Marine broth (Becton Dickinson, 40.1), tryptic soy broth (Becton Dickinson, 13.3), NaCl (30.0), agar (10.0). The pH was adjusted to 7.5. Incubations were carried out in a shaker (200 rpm) at 30 °C and optical density (OD) at 600 nm was determined.

## Supplementary information

Supplementary Information
