# Supplementary material for: Brevibacterium from Austrian hard cheese harbor a putative histamine catabolism pathway and a plasmid for adaptation to the cheese environment
Source: Sci Rep. 2019 Apr 16;9:6164. doi: 10.1038/s41598-019-42525-y (PMC6467879; doi:10.1038/s41598-019-42525-y)
Supplement: Supplementary file 1 — Supplementary Information [file 41598_2019_42525_MOESM1_ESM.pdf]

Supplementary Information

Brevibacterium from Austrian hard cheese harbor a putative histamine catabolism pathway and a plasmid for adaptation to the cheese environment

Justin M. Anast[1,2], Monika Dzieciol[3], Dylan L. Schultz[4], Martin Wagner[3,5], Evelyne Mann[3], Stephan Schmitz-Esser[1,2]*

Affiliations:
[1]Interdepartmental Microbiology Graduate Program Iowa State University, Ames, IA, USA;
[2]Department of Animal Science, Iowa State University, Ames, IA, USA;
[3]Institute for Milk Hygiene, University of Veterinary Medicine Vienna, Vienna, Austria;
[4]Interdepartmetal Microbiology Undergraduate Program, Iowa State University, Ames, IA, USA
[5]Austrian Competence Center for Feed and Food Quality, Safety and Innovation (FFoQSI), Technopark C, 3430 Tulln, Austria.

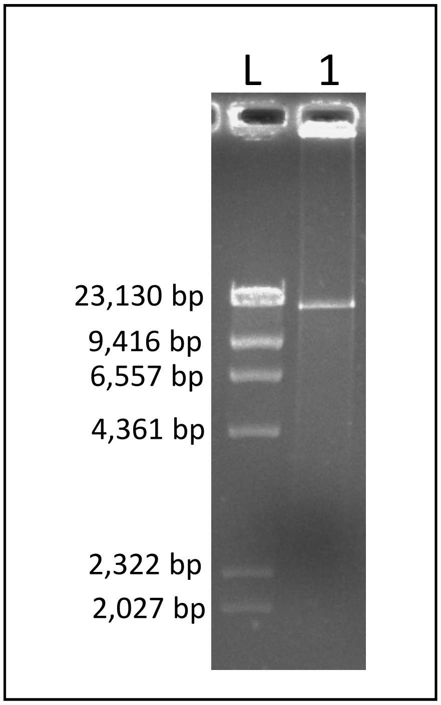

Figure S1. Agarose gel (1% TBE) electrophoresis of pBS22 linearized with HindIII.
Lane 1: linearized product of pBS22 digested with HindIII.
L: Lambda DNA / HindIII Marker

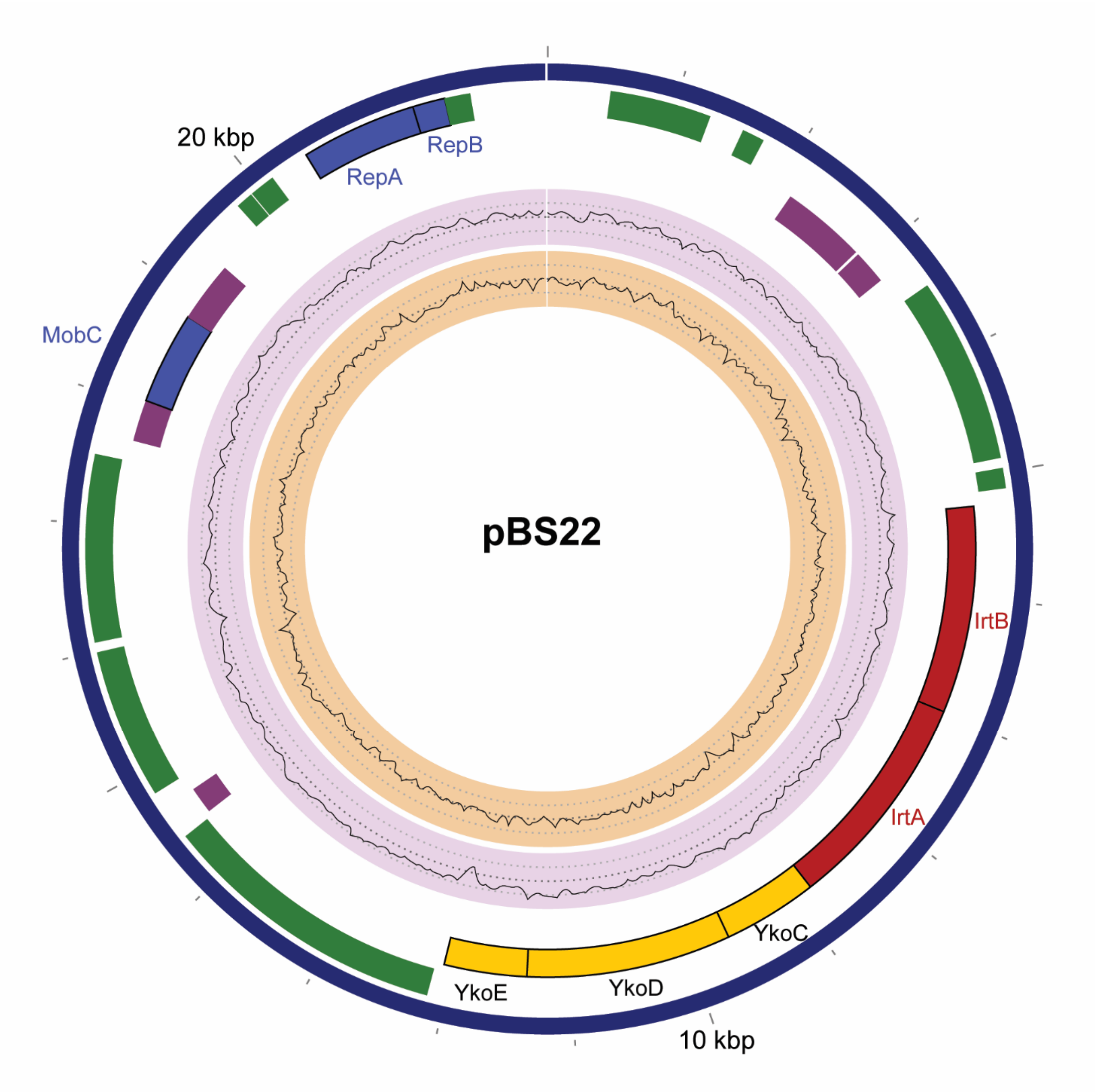

Figure S2. Map of plasmid pBS22.
The map was generated with PATRIC (https://www.patricbrc.org/). Coding sequences on the forward and reverse strand are shown in green and purple, respectively. Replication-associated genes are highlighted in blue, the putative iron transporter IrtAB is highlighted in red and the putative hydroxylmethyl pyrimidine (HMP)/thiamine ABC transporter is highlighted in yellow. The GC content is shown in the lavender ring and the GC skew in beige.

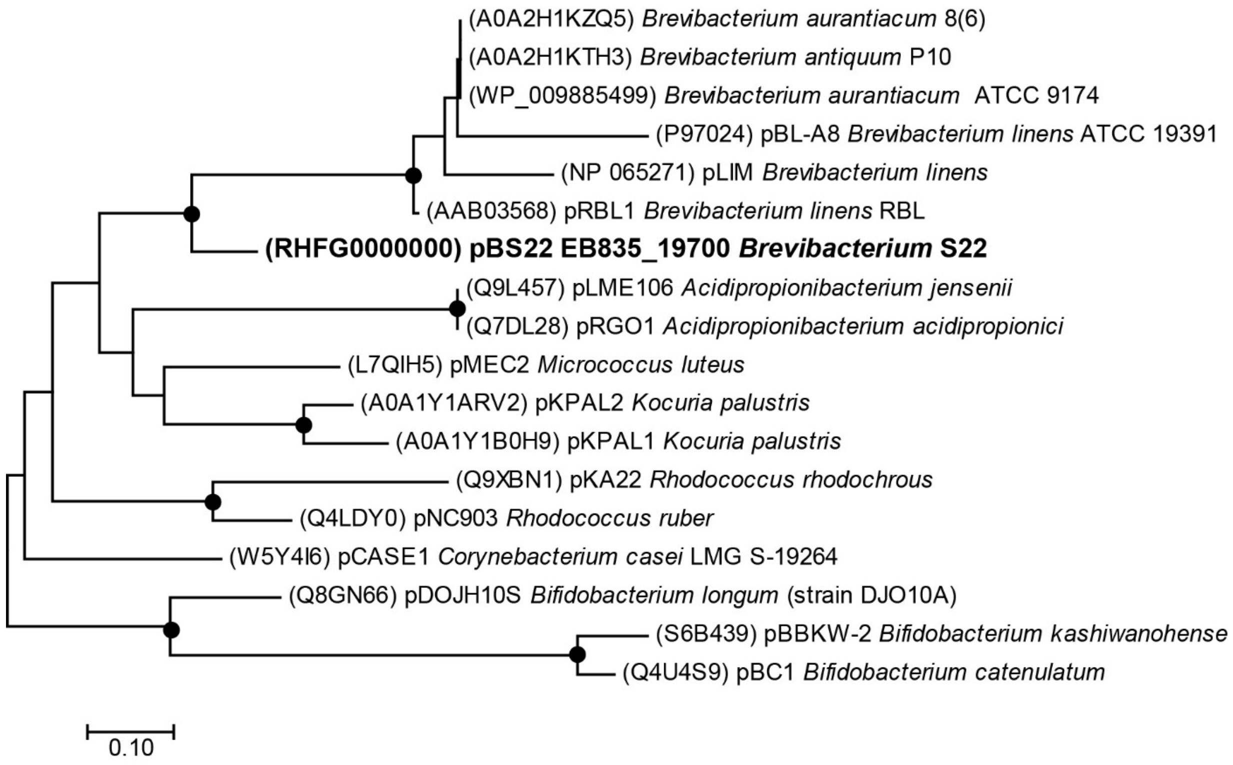

Figure S3. Phylogenetic relationships of RepA plasmid replication protein amino acid sequences from various *Actinobacteria*. The evolutionary history was inferred by using the Maximum Likelihood method based on the Tamura-Nei model. The tree with the highest log likelihood (-5051.74) is shown. Initial tree(s) for the heuristic search were obtained automatically by applying Neighbor-Join and BioNJ algorithms to a matrix of pairwise distances estimated using the Maximum Composite Likelihood (MCL) approach, and then selecting the topology with superior log likelihood value. The tree is drawn to scale, with branch lengths measured in the number of substitutions per site. The analysis involved 18 amino acid sequences. All positions containing gaps and missing data were eliminated. There were a total of 273 positions in the final dataset. Evolutionary analyses were conducted in MEGA7 (Kumar S., Stecher G., and Tamura K. (2016). Molecular Biology and Evolution 33:1870-1874)
Brevibacterium S22 is highlighted in bold. GenBank accession numbers are shown in brackets. Black dots indicate Maximum Likelihood, Neighbor-Joining and Maximum Parsimony bootstrap values higher than 95 (1000x ×resampling).

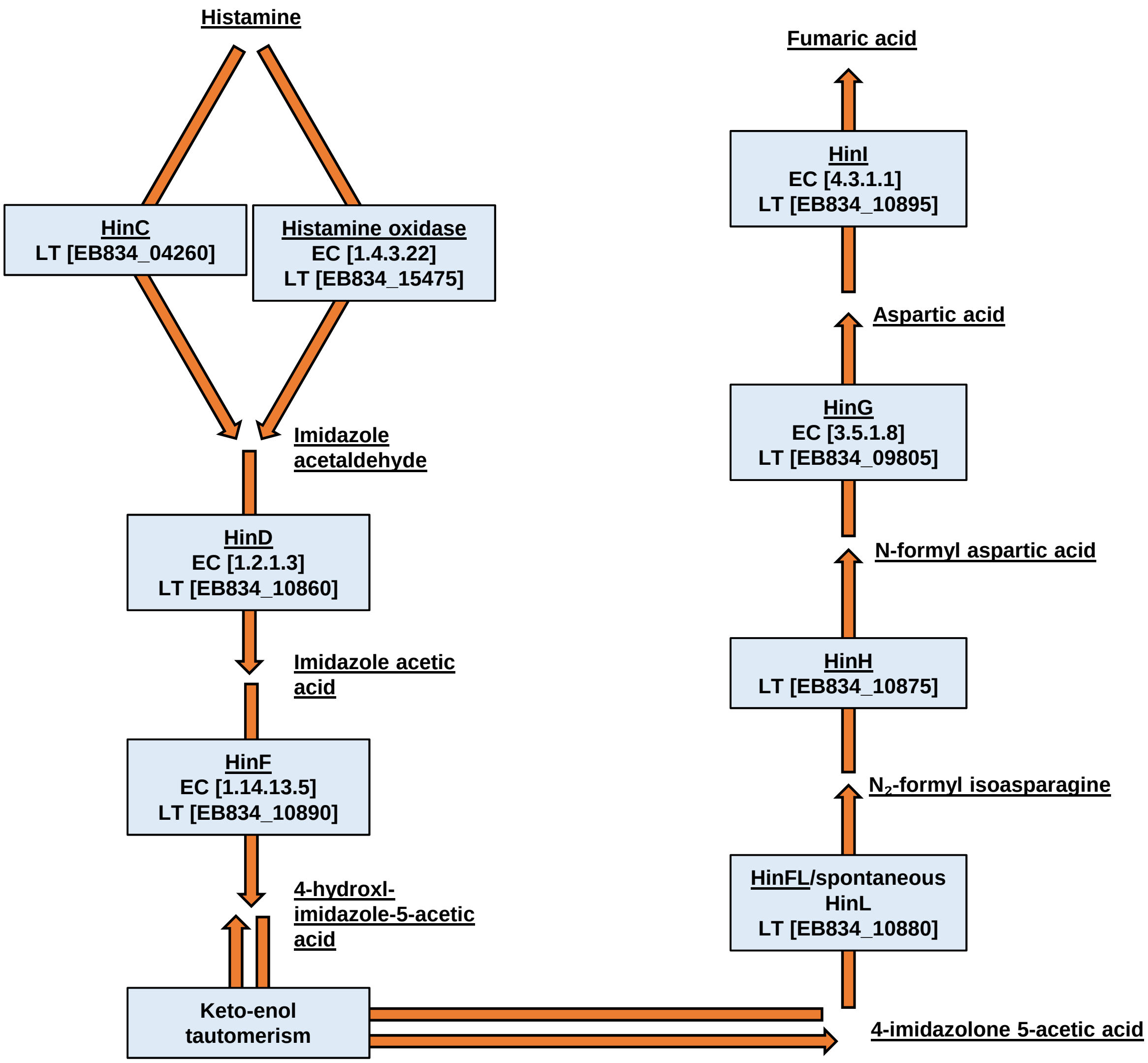

Figure S4. Diagram of the putative histamine catabolism pathway of *Brevibacterium* isolates L261, S111, and S22. The pathway is based on the histamine catabolism pathway described for *Pseudomonas putida* by de la Torre et al. (Environ Microbiol. 2018;20(5):1828-1841). EC, enzyme commission number; LT, locus_tag corresponding to isolate *Brevibacterium aurantiacum* L261 Genbank assignment.

Table S1. ***Brevibacterium*** **bacterial cell equivalents (BCEs) of two cheese production facilities at day 0, 14, 30, 90, and 160 of ripening**

Cheese production plant	Ripening days	Median	Min	Max	IQR[a]
A	0	1.46E+06	4.45E+05	1.52E+07	2.57E+06
	14	2.19E+06	1.50E+06	3.48E+07	1.51E+06
	30	6.18E+06	2.50E+06	3.02E+07	6.57E+06
	90	5.41E+06	3.17E+06	1.16E+07	2.31E+06
	160	5.63E+06	3.70E+06	6.22E+07	2.24E+06
B	0	4.21E+07	1.76E+07	1.32E+08	2.15E+07
	14	6.85E+07	7.07E+06	4.55E+08	5.86E+07
	30	9.08E+06	3.46E+06	1.24E+08	4.31E+07
	90	5.83E+07	8.25E+06	1.08E+08	6.34E+07
	160	1.26E+07	5.94E+06	5.87E+07	1.61E+07

[a]: IQR Interquartile range

Table S2. ***Brevibacterium*** **BCEs comparisons of different ripening times**

Comparison ripening days	0 vs.14	0 vs. 30	14 vs. 30	30 vs. 90	90 vs.160
p-value plant A	0.39	<0.01	<0.01	<0.05	0.47
p-value plant B	<0.05	0.1	<0.05	0.16	<0.01

Table S3: Tetranucleotide correlation analyses of *Brevibacterium* genomes.
Tetranucleotide correlations were calculated using the JSpeciesWS webserver

	B. aurantiacum SMQ-1335	***B. aurantiacum* L261**	***Brevibacterium* S22**	***Brevibacterium* S111**	*B. antiquum* CNRZ 918	*B. aurantiacum* BL2, ATCC 9174	*B. aurantiacum* ATCC 9175	*B. casei* CIP 102111	*B. linens* ATCC 9172	*B. iodinum* ATCC 49514
B. aurantiacum SMQ-1335		**0.9991**	**0.9767**	**0.9819**	0.9972	0.9994	0.9992	0.9313	0.9769	0.9734
***B. aurantiacum* L261**	**0.9991**		**0.9793**	**0.9838**	**0.9969**	**0.9992**	**0.9990**	**0.9371**	**0.9795**	**0.9759**
***Brevibacterium* S22**	**0.9767**	**0.9793**		**0.9937**	**0.9752**	**0.9774**	**0.9790**	**0.9613**	**0.9965**	**0.9962**
***Brevibacterium* S111**	**0.9819**	**0.9838**	**0.9937**		**0.9817**	**0.983**	**0.9831**	**0.9601**	**0.9945**	**0.9927**
B. antiquum CNRZ 918	0.9972	**0.9969**	**0.9752**	**0.9817**		0.9976	0.9972	0.9339	0.9757	0.9720
B. aurantiacum BL2, ATCC 9174	0.9994	**0.9992**	**0.9774**	**0.983**	0.9976		0.9992	0.9327	0.9777	0.9743
B. aurantiacum ATCC 9175	0.9992	**0.9991**	**0.9790**	**0.9831**	0.9972	0.9992		0.9333	0.9787	0.9760
B. casei CIP 102111	0.9314	**0.9371**	**0.9614**	**0.9601**	0.9339	0.9327	0.9333		0.9654	0.9635
B. linens ATCC 9172	0.9769	**0.9795**	**0.9965**	**0.9945**	0.9757	0.9777	0.9787	0.9654		0.9985
B. iodinum ATCC 49514	0.9734	**0.9759**	**0.9962**	**0.9927**	0.9720	0.9743	0.9760	0.9635	0.9985	

Table S4. NCBI Genbank locus_tags of homologs of cheese ripening enzymes found in *Brevibacterium* isolates L261, S111, and S22

	***Brevibacterium* L261**	***Brevibacterium* S111**	***Brevibacterium* S22**
L-methionine gamma-lyase,<br><br>Dias et al. *Appl Environ Microbiol* 64, 3327-3331 (1998)	EB834_06360	EB836_16685	EB835_09420
Proline iminopeptidase,<br><br>Gilbert et al. *Microbiol* 140, 537-542 (1994).	EB834_12345	EB836_08895	EB835_07310
Xaa-Pro aminopeptidase,<br><br>Nardi et al. *Appl Environ Microbiol* 57, 45 (1991)	EB834_08715	EB836_06770	EB835_00415
Cell wall-associated protease,<br><br>Rattray et al. *Appl Environ Microbiol* 61, 3454-3456 (1995)	EB834_09040	EB836_05525	EB835_00735
Aminopeptidase II,<br><br>Fernández et al. *Int Dairy J* 10, 241-248 (2000)	EB834_17750	EB836_12390	EB835_04215
N-terminal sequence of aminopeptidase,<br><br>Rattray et al. *Appl Environ Microbiol* 63, 2468-2471 (1997)	EB834_13725	EB836_01380	EB835_08520
Intracellular esterase,<br><br>Rattray et al. *Int Dairy J* **7**, 273-278 (1997)	EB834_02950	EB836_01495	EB835_18445

Table S5: Amino acid identities of *Brevibacterium* plasmid replication-associated proteins

	Homolog of: amino acid identity, (Genbank accession number), locus_tag					
	**RepA pLIM** (NP_065271.1)	**RepB pLIM** (NP_065272.1)	**ORFIII pLIM** (AAF89087.1)	**RepA pBLA8** (CAA72653.1)	**ORFIII pBLA8** (CAA72654.1)	**RepA pRBL1** (AAB03568.1)
Brevibacterium S22 pBS22	74% EB385_19700	63% EB835_19705	Not detected	68% EB385_19700	Not detected	73% EB835_19700
B aurantiacum ATCC 9174	95% (WP_009885499)[a] BLIN_RS22825	68% (WP_081448679)[a] BLIN_RS23725	89% (WP_009885497)[a]	83% (WP_009885499)[a] BLIN_RS22825	98% (WP_009885497)[a] BLIN_RS23725	95% (WP_009885499)[a] BLIN_RS22825
B aurantiacum 8 (6)	95% (SMY05207)[b] BAURA86_03990	68% (SMY05208)[b] BAURA86_03991	89% (SMY05202)[b] BAURA86_03988	84% (SMY05207)[b] BAURA86_03990	99% (SMY05202)[b] BAURA86_03988	95% (SMY05207)[b] BAURA86_03990
B. antiquum P10	95% (SMY02844)[c] BANT10_03446	68% (SMY02840)[c] BANT10_03445	89% (SMY02852)[c] BANT10_03448	84% (SMY02844)[c] BANT10_03446	99% (SMY02852)[c] BANT10_03448	95% (SMY02844)[c] BANT10_03446
B. linens 947_7	95% (PCC47613)[d] CIK64_04535	68% (PCC47614)[d] CIK64_04540	89% (PCC47612)[d] CIK64_04530	83% (PCC47613)[d] CIK64_04535	99% (PCC47612)[d] CIK64_04530	95% (PCC47613)[d] CIK64_04535

[a] contig: 2662183_Cont197 (NZ_AAGP01000068), 8.7kb
[b] contig: Contig1030 (FXZI01000030), 7.4kb
[c] contig: Contig131 (FXZE01000031), 9.0kb
[d] contig: Contig 107 (NRGP01000006), 22.7kb

Table S6: Amino acid identity and coverage between *Pseudomonas putida* and *Brevibacterium* histamine degradation genes

***P. putida* Hin gene**	***B. aurantiacum* L261**	***Brevibacterium* S111**	***Brevibacterium* S22**	***B. aurantiacum* SMQ-1335**	***B. aurantiacum* ATCC 9174**	***B. aurantiacum* ATCC 9175**	***B. antiquum* CNRZ 918**	***B. casei* CIP 102111**	***B. linens* ATCC 9172**	***B. iodinum* ATCC 49514**
HinA	50 [98]	51 [98]	50 [98]	50 [98]	50 [98]	50 [98]	36 [93]	50 [98]	51 [98]	41 [95]
HinB	Absent	Absent	Absent	Absent	Absent	Absent	Absent	Absent	Absent	Absent
HinC	32 [92]	36 [94]	33 [92]	31 [92]	32 [92]	32 [92]	31 [92]	32 [92]	32 [92]	33 [92]
HinD	50 [98]	50 [99]	50 [99]	44 [96]	44 [96]	44 [96]	39 [95]	50 [99]	44 [96]	38 [95]
HinE	Absent	Absent	Absent	Absent	Absent	Absent	Absent	Absent	Absent	Absent
HinF	63 [92]	60 [94]	61 [92]	Absent	Absent	Absent	Absent	61 [97]	Absent	Absent
HinG	43 [98]	42 [98]	40 [97]	39 [72]	39 [72]	43 [98]	Absent	Absent	42 [97]	41 [97]
HinH	48 [100]	50 [100]	48 [100]	Absent	Absent	Absent	Absent	49 [100]	Absent	Absent
HinI	56 [98]	56 [96]	54 [97]	Absent	Absent	Absent	Absent	54 [96]	54 [96]	56 [96]
HinJ	Absent	Absent	Absent	Absent	Absent	Absent	Absent	Absent	Absent	Absent
HinK	36 [99]	33 [99]	36 [99]	32 [99]	32 [99]	35 [97]	32 [98]	33 [97]	33 [98]	33 [98]
HinL	65 [98]	61 [99]	64 [98]	Absent	Absent	Absent	Absent	63 [98]	Absent	Absent
Histamine Oxidase* [EC:1.4.3.22]	57 [96]	52 [92]	59 [94]	57 [96]	57 [96]	57 [96]	56 [96]	58 [94]	Absent	Absent

Values are represented as: percent amino acid identity [percent query coverage]. Similarities below 30% identity and 70% coverage were considered as absent

* Homolog of the *Arthrobacter globiformis* Histamine Oxidase

Table S7: MIQE guidelines for qPCR.

Item	Importance	Status	Remarks
**Experimental design**			
Definition of experimental and control groups	E[1]	✓	Two cheese diary plants, A and B
Number within each group	E	✓	Number of samples within each group (n=100), number of subgroups (n=5): ripening days 0; 14; 30; 90; 160
Assay carried out by core lab or investigator's lab?	D[2]	✓	Investigator's lab
Acknowledgement of authors' contributions	D	✓	See main manuscript
**Sample**			
Description	E	✓	DNA isolated from cheese rinds
Volume/mass of sample processed	D	✓	250 mg pellet of the homogenized cheese rind sample in duplicate
Microdissection or macrodissection	E	✓	Not relevant
Processing procedure	E	✓	DNA isolation using PowerSoil™ DNA Isolation kit
If frozen - how and how quickly?	E	✓	Samples were stored on ice during transport to the laboratory and processed immediately. After DNA isolation, samples were frozen within 10 minutes at -80°C.
If fixed - with what, how quickly?	E	✓	Samples were not fixed
Sample storage conditions and duration	E	✓	Cheese rind samples were processed immediately. DNAs were stored 1-6 months at -80°C, after thawing on ice, DNA samples were applied to the qPCRs within 5min
**Nucleic acid extraction**			
Procedure and/or instrumentation	E	✓	PowerSoil™ DNA Isolation kit, mechanical lysis
Name of kit and details of any modifications	E	✓	PowerSoil™ DNA Isolation kit (MoBio Laboratories, Carlsbad, CA, USA), no modifications
Source of additional reagents used	D	✓	DNA was eluted in DEPC-treated water
Details of DNase or RNase treatment	E	✓	No treatment
Contamination assessment (DNA or RNA)	E	✓	NTCs included to DNA isolation were analyzed with qPCR
Nucleic acid quantification	E	✓	Qubit® 2.0 Fluorometer
Instrument and method	E	✓	Qubit® 2.0 Fluorometer (Thermo Fisher Scientific, Vienna, Austria)
Purity (A_{260}/A_{280})	D	✓	
Yield	D	✓	
RNA integrity method/instrument	E		Not relevant
RIN/RQI or Cq of 3' and 5' transcripts	E		Not relevant
Electrophoresis traces	D	✓	Aliquots of qPCR products were analyzed by agarose gel electrophoresis
Inhibition testing (Cq dilutions, spike or other)	E	✓	No inhibition determined (dissociation curves, gel electrophoresis analysis, specificity of the amplicons verified by DNA sequencing of the PCR products)
**Reverse transcription**			
Reaction conditions	E		Not relevant
Amount of RNA and reaction volume	E		Not relevant
Priming oligonucleotide and concentration	E		Not relevant
Reverse transcriptase and concentration	E		Not relevant
Temperature and time	E		Not relevant
Manufacturer of reagents and catalogue numbers	D		Not relevant
Cqs with and without RT	D		Not relevant
Storage conditions of cDNA	D		Not relevant
**qPCR target information**			
Gene symbol	E	✓	16S rRNA gene
Sequence accession number	E	✓	*Brevibacterium* sp. S22 partial 16S rRNA gene, isolate S22 – Locus_tag: EB835_20185; Accession: RHFG00000000<br>*Brevibacterium* sp. S111 partial 16S rRNA gene, isolate S111 – Locus_tag: EB836_18025; Accession: RHFH00000000<br>*Brevibacterium* sp. L261 partial 16S rRNA gene, isolate L261 – Locus_tag: EB834_20190; Accession: RHFF00000000
Location of amplicon	D	✓	
Amplicon length	E	✓	16S rRNA gene genus *Brevibacterium* - 125bp
In silico specificity screen (blast, etc)	E	✓	Ribosomal Database Project (RDP) probe match tool, Primer3, NCBI primer designing tool, TestPrime arb-SILVA

Pseudogenes, retropseudogenes or other homologs?	D		Not relevant
Sequence alignment	D		Not relevant
Secondary structure analysis of amplicon	D	✓	
Location of each primer by exon or intron (if applicable)	E		Not relevant
What splice variants are targeted?	E		Not relevant
**qPCR oligonucleotides**			
Primer sequences	E	✓	See main manuscript
RTPrimerdb identification number	D	✓	Not done, beside 16S rRNA gene for *Brevibacterium*, all of them are unpublished newly designed primers
Probe sequences	D	✓	Not done, as no probes were used
Location and identity of any modifications	E	✓	No modifications
Manufacturer of oligonucleotides	D	✓	Microsynth
Purification method	D	✓	Desalted
**qPCR protocol**			
Complete reaction conditions	E	✓	See main manuscript
Reaction volume and amount of cDNA/DNA	E	✓	25 µl reaction volume (incl. 5 µl DNA)
Primer, (probe), Mg++ and dNTP concentrations	E	✓	See main manuscript
Polymerase identity and concentration	E	✓	1.5U of Platinum® Taq DNA polymerase (Thermo Fisher Scientific, Vienna, Austria)
Buffer/kit identity and manufacturer	E	✓	See main manuscript
Exact chemical constitution of the buffer	D	✓	10×PCR Buffer, – Mg; (Invitrogen, Vienna, Austria)
Additives (SYBR green I, DMSO, etc.)	E	✓	no further additives
Manufacturer of plates/tubes and catalog number	D	✓	MicroAmp optical tube (0.2 µl; Applied Biosystems by life technologies)
Complete thermocycling parameters	E	✓	94°C for 2 min and 45 cycles of 94°C for 30 s followed by 60 s at 60°C, melting curve 50°C to 90°C
Reaction setup (manual/robotic)	D	✓	Manual
Manufacturer of qpcr instrument	E	✓	Stratagene Mx3000P real-time PCR System (Agilent Technologies, Santa Clara, USA)
**qPCR validation**			
Evidence of optimisation (from gradients)	D	✓	Concentrations of primers in range of 200-400 nM, $MgCl_2$ ranging from 2 to 3.5 mM, as well as annealing/extension temperature, ranging from 60°C to 64°C were tested
Specificity (gel, sequence, melt, or digest)	E	✓	Gel, sequence, melting curve
For SYBR green, Cq of the NTC	E	✓	No amplification
Standard curves with slope and y-intercept	E	✓	Done
PCR efficiency calculated from slope	E	✓	98.7%
Confidence interval for PCR efficiency or standard error	D		-
r^2 of standard curve	E	✓	Between 0.997 and 1 for all primer pairs
Linear dynamic range	E	✓	Determined, 7 log scales tested
Cq variation at lower limit	E	✓	Less than 4% within replicates
Confidence intervals throughout range	D	✓	
Evidence for limit of detection (LOD)	E	✓	2.44e+04 BCE per 0.5g cheese rind
If multiplex, efficiency and LOD of each assay.	E		Not relevant, no multiplexing
**Data analysis**			
qPCR analysis program (source, version)	E	✓	Stratagene Mx3000P real-time PCR System (Agilent Technologies, Santa Clara, USA)
Method of Cq determination	E	✓	Stratagene Mx3000P real-time PCR System settings (baseline subtracted curve fit, single threshold, automatically calculated). Threshold manually curated for maximum efficiency within linear range for each plate

Outlier identification and disposition	E	✓	Done
Results of NTCs	E	✓	No amplificates
Justification of number and choice of reference genes	E		Not done
Description of normalisation method	E		Not done
Number and concordance of biological replicates	D	✓	2 biological replicates
Number and stage (RT or qPCR) of technical replicates	E	✓	2 technical replicates for all samples and standards
Repeatability (intra-assay variation)	E	✓	Repeatable
Reproducibility (inter-assay variation, %CV)	D	✓	Not determined (strongly recommended for clinical/diagnostic applications, but not other assays)
Power analysis	D	✓	Done
Statistical methods for result significance	E	✓	Wilcoxon Signed-Rank test
software (source, version)	E	✓	R (version 3.2.5, psych package 1.6.12).
Cq or raw data submission using RDML	D	✓	
